# The Effects of the COVID 19 Pandemic on the Clinic of Inpatients in the Child and Adolescent Psychiatry Service

**DOI:** 10.1192/j.eurpsy.2023.502

**Published:** 2023-07-19

**Authors:** E. Karagöz Tanıgör, S. Alşen Güney, B. Baykara, C. Özgül

**Affiliations:** Child and Adolescent Psychiatry, Dokuz Eylul University Faculty of Medicine, İzmir, Türkiye

## Abstract

**Introduction:**

The COVID-19 pandemic and the measures to control the pandemic adversely affected the mental health of children and adolescents; however, studies examining the effects of the pandemic on child and adolescent mental health services are scarce

**Objectives:**

Our study aims to show how this unexpected pandemic affecting all humanity and the accompanying restrictions affect the admissions to the child psychiatry service.

**Methods:**

In this study, the diagnoses, the treatments used, and the length of hospital stay of inpatient child and adolescent psychiatry patients were obtained and compared between the normal period and the pandemic period. Patients who received inpatient treatment between March 2020 and March 2022 and 154 patients who were hospitalized in the year before the pandemic were compared. The effect of the pandemic on the clinical profile of inpatients in the psychiatry ward was measured. For the psychiatric evaluation of the patients, Kiddie Schedule for Affective Disorders and Schizophrenia was used.

**Results:**

When the drugs used by the patients in the ward were compared, there was a difference between the groups in terms of antipsychotic use. While there was a difference in the use of risperidone and aripiprazole(p<0.05); there was no difference in the use of paliperidone, quetiapine, olanzapine, and clozapine (p>0.05). There was a difference between the groups in terms of antidepressant use. While there was a difference in fluoxetine use; there was no difference between the use of sertraline citalopram and escitalopram(p<0.05). No difference was observed between the use of other drugs, methylphenidate and atomoxetine, anxiolytic use, and the use of mood stabilizers lithium valproate lamotrigine (p>0.05). It is observed that there is an increase in the need for antidepressants and antipsychotics used in the child and adolescent psychiatry service during the Covid 19 pandemic.

**Conclusions:**

These results can help inform and develop strategies and interventions related to the pandemic in children and adolescents. Future research should continue to evaluate the psychological consequences of COVID-19 on adolescents. The treatment patterns that were used seemed to change indicating the pandemic had a significant effects on these patients. However, this statement requires to be backed up by other studies to get a conclusion, especially the ones with higher numbers of subjects and longer durations of follow-ups.

**Disclosure of Interest:**

None Declared

